# *Helicobacter pylori* and gastric carcinoma: potential carcinogen, cancer sentinel, or both?

**DOI:** 10.1054/bjoc.2000.1391

**Published:** 2000-09-04

**Authors:** S Ponce-de-León, J E Leal, R Cortés

**Affiliations:** Departments of Clinical Epidemiology and Surgery, Instituto Nacional de la Nutrición Salvador Zubirán, Vasco de Quiroga 15, México, DF. CP 14000, México


British Journal of Cancer (2000) **83**(7), 969–970
© 2000 Cancer Research Campaign
doi: 10.1054/ bjoc.2000.1391, available online at http://www.idealibrary.com on IDEAL®

Letter to the Editor

### *Helicobacter pylori* and gastric carcinoma: potential carcinogen, cancer sentinel, or both?

**Sir,**

Because *Helicobacter pylori* (HP) is widely accepted as a risk factor for gastric carcinoma (Blaser, 1999; Scheiman and Cutler, 1999), mass eradication has been proposed to prevent this common neoplasm (Scheiman and Cutler, 1999; Danesh, 1999). The postulated causal relationship, however, may be distorted by detection bias in which a 'silent' cancer is coincidentally found when dyspeptic symptoms lead to an earlier diagnostic study. Evidence of this effect is an increased stage ratio of invasive/non-invasive gastric cancer in patients without HP.

During a search of factors associated with presentation stage in 373 patients diagnosed with gastric cancer in the years between December of 1987–1998, we found a higher frequency of HP in biopsies of patients with non-invasive TNM stages I and II (14/49, 28.6%) than in those with invasive TNM stages III–IV (38/324, 11.7%) ($P = 0.0034$). The invasive to non-invasive stage ratio was 6.6:1. This finding was independent of other factors also showing a significant association such as socio-economic status and the interval between the onset of symptoms and diagnosis of cancer.

Although not statistically significant, the interval between symptoms and diagnosis was shorter in patients with *H. pylori* than in those without it (median difference of 26 days, $P > 0.20$). Also, the shortest median interval to diagnosis was in patients with non-invasive stages and HP present, whereas the longest interval was for those with invasive stages and without HP (Table 1). The results support the argument that the cancer is found sooner in those with rather than without HP.

The paradoxical finding of HP, or its antibodies, more often in relatively early stages of gastric cancer than in advanced ones has been previously observed (Caruso and Fucci, 1990). A proposed explanation has been a change in the conditions favouring HP persistence as the tumour grows (Forman et al, 1994), but an alternative explanation is that the association between HP and gastric cancer has been distorted by detection bias, specifically in test ordering. The dyspeptic symptoms caused by the infection lead to an endoscopy, or an X-ray study, in which a still symptomless gastric tumour is found. Thus, in patients with gastric neoplasia, coexistence of HP infection increases the likelihood of going to a physician and having an endoscopy (or a GI X-ray series). Without the infection, the tumour may not be detected until it advances to produce unequivocal symptoms.

**Table 1** Time to diagnosis in gastric cancer according to stage and presence of *Helicobacter pylori*

| Tumour stage | *Helicobacter pylori* | *n* | Median (days) | 95% Confidence Interval (days) |
|---|---|---|---|---|
| Non-Invasive | Present | 13 | 49 | 21.5 to 179 |
| | Absent | 35 | 121 | 55 to 265 |
| Invasive | Present | 38 | 176 | 110.5 to 366.8 |
| | Absent | 285 | 185 | 95 to 324.5 |

The issue is important because several trials are now under way to test the benefits of mass eradication of HP for 'preventing' gastric cancer (Danesh, 1999). The elimination of the infection, however, may remove the benefit of its sentinel role in early detection and may worsen an already high ratio of advanced to non-advanced cases of this common malignancy.

*S Ponce-de-León, J E Leal, R Cortés*
*Departments of Clinical Epidemiology and Surgery, Instituto Nacional de la Nutrición Salvador Zubirán, Vasco de Quiroga 15, México, DF. CP 14000, México*

### REFERENCES

Blaser MJ (1999) In a world of black and white, *Helicobacter pylori* is gray. *Ann Intern Med* **130**: 695–7

Caruso ML and Fucci L (1990) Histological identification of *Helicobacter pylori* in early and advanced gastric cancer. *J Clin Gastroenterol* **12**: 601–602

Danesh J (1999) *Helicobacter pylori* and gastric cancer: time for mega-trials? *Br J Cancer* **80**: 927–929

Forman D, Webb P and Parsonnet J (1994) *H. pylori* and gastric cancer. *Lancet* **343**: 243–244

Scheiman JM and Cutler AF (1999) *Helicobacter pylori* and gastric cancer. *Am J Med* **106**(2): 222–226

